# Enhanced psychosocial assessment and rapid follow-up care for people presenting to emergency departments with self-harm and/or suicidal ideation: the Assured feasibility study and internal pilot trial

**DOI:** 10.1186/s40814-025-01602-y

**Published:** 2025-02-20

**Authors:** Sally O’Keeffe, Mimi Suzuki, Mary Ryan, Stefan Priebe, Richard Byng, Alan Simpson, Vera Araújo-Soares, Rikke Albert, Renata Fialho, Neil Walker, Alexandra Elissavet Bakou, Rose McCabe

**Affiliations:** 1 Newcastle University, Newcastle-Upon-Tyne, UK; 2 City St George’s, University of London, London, UK; 3 Queen Mary, University of London, London, UK; 4 University of Plymouth, Plymouth, UK; 5 King’s College London, London, UK; 6 Heidelberg University, Heidelberg, Germany; 7 East London NHS Foundation Trust, London, UK; 8 Surrey and Borders Partnership NHS Trust, Surrey, UK

**Keywords:** Emergency department, Feasibility study, Liaison psychiatry, Self-harm, Suicide

## Abstract

**Background:**

Patients presenting to emergency departments (EDs) following an episode of self-harm are at risk of future suicide. There are few evidence-based interventions for self-harm in the ED context in England. This study sought to assess the feasibility of a trial of a newly developed brief psychological intervention, the Assured approach. This approach consisted of an enhanced psychosocial assessment, collaborative safety planning and three rapid solution-focussed follow-up sessions. Phase 1 was a feasibility study, and phase 2 was an internal pilot trial of a cluster randomised controlled trial to assess whether progression to a full-scale trial was warranted.

**Methods:**

In phase 1, patients were recruited and allocated to a study arm, the Assured arm or treatment as usual, depending on the allocation of their assessing practitioner, in four EDs in England. They were invited to research assessments after consent and at 6 months. Phase 2 was the internal pilot of a cluster randomised controlled trial conducted in six EDs in England. Practitioners were randomised to deliver the Assured approach or treatment as usual. Patients were recruited and allocated to a study arm depending on the allocation of their assessing practitioner. They were invited to complete research assessments after consent and at 3, 9 and 18 months.

**Results:**

Sixty-one patients were recruited into the Assured (*n* = 46) and treatment as usual (*n* = 15) arms in phase 1. Findings showed we could recruit and follow up patients over a 6-month period. The research procedures were acceptable to patients and practitioners, and the intervention was delivered with acceptable fidelity to the intervention manual. Forty-seven patients were recruited into the phase 2 internal pilot trial, falling substantially short of our target of 491 in the stop-go criteria, indicating that the trial was not feasible in its current design.

**Conclusion:**

The feasibility study indicated that both the intervention and research processes were acceptable. However, the internal pilot trial revealed substantial challenges in recruiting patients and delivering the intervention in the ED context. Adaptations to the trial design and intervention are proposed to enable the Assured approach to be tested in a future trial, to improve care for this underserved population.

**Trial registration:**

ISRCTN16003313, 04/02/2020; ISRCTN13472559, 18/11/2021.

**Supplementary Information:**

The online version contains supplementary material available at 10.1186/s40814-025-01602-y.

## Key messages regarding feasibility

- What uncertainties existed regarding the feasibility?
  It was not clear whether a brief psychological intervention could be delivered in emergency departments in the UK NHS setting with patients following a self-harm/suicidal crisis and whether it was feasible to conduct a randomised controlled trial of this intervention.
- What are the key feasibility findings?
  In the phase 1 pilot study, we successfully recruited patients from emergency departments and followed them up over a 6-month period. However, progression to phase 2 which was an internal pilot trial revealed significant issues with recruitment.
- What are the implications of the feasibility findings for the design of the main study?
  The stop-go criteria for progression to the full-scale randomised controlled trial were not met, due to the significant challenges faced by emergency departments particularly following the COVID-19 pandemic. An amended trial design is proposed to take into account the real-world challenges of conducting a trial in this context that will enable the clinical- and cost-effectiveness of the intervention to be tested.

## Background

Suicide is a leading cause of death worldwide [1]. In the U.K., approximately 6000 people take their own lives each year [2]. The strongest risk factor for suicide is self-harm [3]. Self-harm refers to intentional self-poisoning or self-injury, irrespective of motive or the extent of suicidal intent [4]. It includes acts intended to result in suicide (attempted suicide), those without suicidal intent (e.g. as a coping mechanism) and acts where there is a mixed or unclear motivation. Each year, approximately 220,000 episodes of self-harm by 150,000 people are managed by emergency departments (EDs) in England [5]. One in 25 people who present at the hospital for self-harm die by suicide within the subsequent 5-year period [3], making this a crucial opportunity for intervention.

Most EDs in England have a liaison psychiatry team staffed by specialist mental health practitioners. National Institute for Health and Care Excellent (NICE) guidelines recommend a biopsychosocial assessment by specialist mental health practitioners in the ED for people who present with self-harm [4]. Mental health practitioners conduct biopsychosocial assessments to assess the person’s current and future health and social care needs and make onward referrals. While the rollout of liaison psychiatry teams in EDs provide mental health support for people attending ED in crisis, biopsychosocial assessments for those attending EDs in the UK are inconsistent across services and are often inadequate [6]. Recent studies describe the stigma experienced by many people seeking help for self-harm in EDs [7]. People have emphasised the need for compassion, understanding and hope when seeking help in times of distress, yet the lack of treatment for self-harm in the wider system means many people have no support in place aside from crisis care [7–9].

To improve quality of life, reduce future self-harm and reduce suicide risk, effective ED interventions are needed. Mental healthcare practitioners in EDs are positioned to provide interventions that can be delivered at scale. Evidence from recent international trials indicates that brief, low-cost, psychological interventions delivered by specialist mental health practitioners in EDs are effective in reducing self-harm and suicide [10]. This study sought to pilot a brief psychological intervention in the ED context in England.

Drawing on international evidence [10], focus groups and semi-structured interviews with stakeholders [7], we adapted a brief intervention for people presenting to EDs for self-harm and suicidal ideation for the National Health Service (NHS) context [11], the Assured approach. The intervention consisted of an enhanced psychosocial assessment in the ED to maximise the therapeutic potential of routine ED contacts, safety planning and solution-focussed follow-up sessions over 8 weeks. This differs from standard care which focusses on risk assessment/management, safety plans which focus on professional support (e.g. crisis line numbers), and typically, there is no follow-up care after the person leaves the ED. The Assured approach therefore seeks to maximise the therapeutic potential of the ED assessment and provide rapid follow-up care in the weeks following discharge from the ED. This study investigated the feasibility of testing the intervention in a future randomised controlled trial (RCT) in NHS EDs in England.

### Objectives

The study had two phases. Phase 1 was a feasibility study that followed the Medical Research Council (MRC) framework to design and evaluate complex interventions [12]. We designed a non-randomised study. The main aim was to test the feasibility of evaluating an intervention for patients presenting to the ED with self-harm and/or suicidal ideation. To assess feasibility, phase 1 had six specific objectives:

1. To test the feasibility of recruiting patients presenting to the ED with self-harm and/or suicidal ideation to a study testing a brief psychological intervention;
2. To pilot patient outcome measures;
3. To pilot collection of practitioner-report data;
4. To explore patients’ experiences of taking part in the study;
5. To explore patients’ engagement in the intervention;
6. To explore fidelity to the intervention manual.

Phase 2 was an internal pilot trial in a cluster randomised controlled trial. The main aim was to assess whether the stop-go criteria were met to progress to a full-scale trial.

## Phase 1: Methods

### Design

The study took place in participating EDs, where we recruited practitioners working in the ED liaison psychiatry team. Practitioners were assigned to deliver treatment as usual (TAU) or the intervention. As this was a feasibility study, testing practitioner randomization was not one of the objectives, so the assignment was based on interest in delivering the intervention.

### Participants

We recruited NHS practitioners working in liaison psychiatry teams (e.g. mental health nurses, psychiatrists and psychologists) to take part in the study from four EDs in Southeast England. Practitioners who consented to take part were assigned to deliver the intervention or TAU; those assigned to the intervention arm received 2 days of training in the intervention.

Patients (16 years or over) presenting to an ED with self-harm, defined as an intentional act of self-poisoning or self-injury, irrespective of the motivation or apparent purpose of the act, or suicidal ideation, were recruited. Exclusion criteria were admission to a psychiatric hospital, cognitive (e.g. dementia) or other psychiatric difficulties interfering with the ability to participate, experiencing a psychotic episode, having no capacity to provide written informed consent, needing an interpreter, Ministry of Justice patients subject to a restriction order and receiving intensive psychological input that precluded receiving another psychological intervention (e.g. dialectical behaviour therapy). While patients aged 16 and over were included, in practice, the liaison psychiatry teams in most of the sites only see patients aged over 18.

As this was a feasibility study, the target number of participants was not determined by a hypothesis-driven sample size calculation. We aimed to recruit 20 practitioners in total (5 TAU and 15 intervention) and 60 patients, with approximately a 1:4 ratio in the TAU: intervention arm. The difference in sample sizes was due to the requirement for a larger sample in the intervention arm to adequately test and refine the intervention, whereas a smaller sample size in TAU was sufficient to explore whether the recruitment processes were suitable for future RCT.

### Intervention

#### The Assured intervention

We developed the Assured intervention for the ED context in the UK. To do this, a systematic review was conducted to investigate the international evidence base for brief interventions for suicidal presentations, which identified components of effective interventions i.e. therapeutic engagement, information provision, safety planning and follow-up contacts [10]. These components formed the basis of the draft intervention. Stakeholders were consulted with the draft intervention in focus groups and semi-structured interviews. These were conducted with patients with experience of presenting to the ED with self-harm, carers of such patients, liaison psychiatry and ED practitioners, and feedback was used to refine the intervention. Further detail on the intervention development process has been published elsewhere [11]. The final intervention consisted of four components:

1. Enhanced psychosocial assessment: In the biopsychosocial assessment in the ED, practitioners were trained in therapeutic techniques to maximise the therapeutic potential of the assessment. This consisted of:
  - Narrative interview: The practitioner started the assessment with a narrative interview. This invited the patient to tell their story leading up to the crisis. This process followed the principles of narrative interviewing from the Attempted Suicide Short Intervention Program (ASSIP) intervention [13]. The narrative interview consisted of encouraging the patient to elaborate on their story, validating their distress and instilling hope. The narrative interview sought to develop a therapeutic alliance to help patients feel listened to, believed, hopeful and supported. The narrative approach allows for NICE-compliant self-harm assessments, with the aim of conducting the assessment in a more patient-centred way.
  - Safety plan: At the end of their biopsychosocial assessment, the practitioner worked with the patient to develop a personalised and enhanced safety plan, based on Stanley & Brown’s safety planning intervention [14]. The safety plan was co-produced to identify the patient’s warning signs, internal and external coping strategies, and informal and formal support networks to improve awareness and self-management of future self-harm. The practitioner worked with the patient to identify barriers to the use of these strategies and steps to overcome the barriers, to maximise the potential for the use of the safety plan in future crises.
2. Check-in phone call: Within 72 h of leaving the emergency department, the practitioner made a check-in phone call to remind the patient of their upcoming follow-up session.
3. Follow-up sessions: The patient was then offered three follow-up sessions over a 2-month period with the same practitioner they had their enhanced psychosocial assessment and check-in phone call with. These sessions used a solution-focussed approach [15], at approximately 1, 4 and 8 weeks after the enhanced psychosocial assessment. The practitioner worked with the patient to explore their future hopes and identify the possible resources and strengths already present in achieving or working towards these. Focusing on the patient’s own hopes, strengths and solutions, rather than problems, aimed to build on what was working in their life [16].
4. Letters: At 3, 6 and 9 months, the patient received personalised letters from the practitioner to remind them of the safety plan and support networks.

The Assured approach was offered in addition to any other support that the patient would be offered e.g. referrals to other services, as described in TAU. This approach differs from standard care in the ED which focusses on risk assessment and management, care plans which generally focus on professional support (e.g. crisis line numbers) and no follow-up from ED.

#### Treatment as usual

TAU consisted of a biopsychosocial assessment as defined by NICE guidelines [4]. This could include discharge to primary care and/or referral to another service e.g. community mental health services or home treatment team, depending on the need.

### Training in the Assured approach

Practitioners allocated to deliver the Assured intervention received a 2-day training in the intervention. This included an introduction to each component of the intervention (narrative interviewing, safety planning and solution focussed practice), watching videos of these techniques being used, discussion and role play to practice the techniques. Top-up training was provided as a refresher for practitioners who were trained prior to the study being paused due to the pandemic, in advance of them delivering the intervention once recruitment opened at their sites. Practitioners were provided with regular supervision to support their delivery of the intervention.

### Procedures

#### Practitioner recruitment and data collection

Practitioners were approached about the study via the team manager. Written consent was obtained, and practitioners were then asked to complete a sociodemographic questionnaire and a validated measure of staff burnout measure: the Maslach Burnout Inventory [17]. The MBI consists of 22 items that are scored using frequency ratings from 0 (never) to 6 (every day). The MBI has three component scales: emotional exhaustion (scores range from 0 to 63 with higher scores indicating higher levels of emotional exhaustion), personal achievement (scores range from 0 to 56 with higher scores indicating higher levels of personal achievement) and depersonalization (scores range from 0 to 35 with higher scores indicating higher levels of depersonalization). The MBI is a reliable and valid measure of work-related burnout. Convergent and discriminant validity of the MBI has been demonstrated, and moderate to high reliability on the subscales has been reported for emotional exhaustion (Cronbach’s *α* = 0.90), personal achievement (Cronbach’s *α* = 0.79) and depersonalisation subscales (Cronbach’s *α* = 0.71) [18, 19].

#### Patient recruitment and data collection

Patients presenting to the ED with self-harm and/or suicidal ideation were recruited. The study ran from February 2020 to November 2021, which meant the study procedures had to be adapted due to the COVID-19 pandemic. For this reason, recruitment was paused between March 2020 and November 2020. Due to the pandemic, there were two recruitment pathways. Following presentation to the ED, patient consent took place via either:

1. Consent in the ED with an on-site researcher. This approach, planned prior to the COVID-19 outbreak, involved researchers being on-site to recruit patients. The patient was referred to the liaison psychiatry team for a biopsychosocial assessment following a presentation to the ED for self-harm and/or suicidal ideation. After the referral to liaison psychiatry, the practitioner asked the patient if they were interested in speaking to a researcher about the study. With the patient’s agreement, the researcher provided the patient with the Participant Information Sheet (PIS) and obtained written consent. The researcher then left and the practitioner conducted the biopsychosocial assessment.
2. Remote consent. This approach was introduced during the pandemic, when researchers were not permitted to be on site, between March 2020 and September 2021. Recruitment procedures were adapted whereby the liaison psychiatry practitioner obtained verbal consent from the patient to take part in the study at the beginning of the biopsychosocial assessment. After the biopsychosocial assessment, the practitioner then passed on the patients’ contact details to the research team. A researcher contacted the patient in the following days, provided them with the PIS and obtained written consent via a secure electronic database.

Patient allocation to a study arm was based on the participating practitioner they were referred to who was responsible for conducting their assessment i.e. whether the practitioner was trained in the Assured approach (intervention arm) or not (TAU). Based on the principle of equipoise [20], patients were informed that practitioners were trained in different ways of conducting assessments and follow-up care, and they would use the approach they had been trained in.

The follow-up period was 6 months. Two research assessments were undertaken with patients. The first part of the intervention was conducted during the biopsychosocial assessment, which took place in the ED within a 1-h referral window from the ED to the liaison psychiatry team. Due to the way participants presented to the study, via ED departments, it was not possible to obtain baseline data prior to the biopsychosocial assessment. Thus, the first research assessment took place as soon as possible after the biopsychosocial assessment, ideally within 1 week. The second research assessment took place 6 months after the study participation consent. Patients were given the option of completing the research assessments in person (subject to COVID-19 restrictions) or over the phone/video call. In addition, patients were provided with the option of completing self-report measures via an electronic database.

Patients also received a survey, each month for 6 months following consent, asking about self-harm episodes over the past month. They received a link via email which directed them to a brief online survey, which was devised for this study.

#### Proposed primary efficacy outcome

The proposed primary efficacy outcome for the future RCT was repeat self-harm resulting in re-presentation to the ED at 6 months. The 6-month follow-up period was dictated by the funding. In phase 1, we piloted identifying these data by searching local ED electronic records, using the gold standard approach developed in the Multicentre Study of Self-Harm in England [21]. Repeat hospital presentation for self-harm was coded as a binary outcome (reattended (not regarding the actual number of attendances) or did not reattend).

#### Secondary outcomes

We measured the following:

1. Self-reported self-harm, obtained using a monthly survey sent via email, sent monthly over the 6-month follow-up period (Appendix 1). This provided a binary response as to whether they self-harmed in the past month (i.e. self-harmed in the past month or did not self-harm in the past month). This was a measure developed for this study by the Lived Experience Advisory Panel (LEAP).
2. Death by suspected suicide, ascertained from medical electronic records.
3. The therapeutic relationship, self-rated by patients on the Helping Alliance Scale [22], adapted for this study, assessed at both research assessments. This scale was adapted specifically for this study to provide a questionnaire that was suitable for rating the therapeutic relationship for single healthcare contacts. It consisted of five items rated on a scale ranging from 0 to 10 and one categorical that asked about how they felt after meeting with the practitioner.
4. Suicidality, administered by researchers on the Columbia–Suicide Severity Rating scale (C-SSRS) [23], assessed at both research assessments. Scores are used to derive three outcomes: suicidal ideation, intensity of ideation and suicidal behaviour. Suicidal ideation is a binary outcome (present or not present). Suicidal intensity is an ordinal outcome, with scores ranging from 2 to 25, with higher scores indicating more intense ideation. Suicidal behaviour is a binary outcome (present or not present). The measure demonstrated good convergent and divergent validity with other measures of suicidal ideation and behaviour [23].
5. Quality of life measured with the Clinical Outcomes in Routine Evaluation – Outcome Measure (CORE-OM) [24], assessed at both research assessments. The mean score of the items ranges from 0 to 4, with higher scores reflecting poorer functioning. Studies have demonstrated good reliability and convergent validity of the measure [25].
6. Social outcomes, measured with the Social Outcomes Index (SIX) [26], assessed at both research assessments. This assesses four domains (employment, accommodation, partnership/family and friendship), and the total score ranges from 0 to 6, with higher scores indicating a better social situation. It is based on objective indicators that can be reliably assessed and has been demonstrated to capture change over time [26].
7. Quality of life, measured with the Manchester Short Assessment of Quality of Life [27], assessed at both research assessments. The score is calculated as the mean of 12 items scored from 1 to 7, with higher scores indicating better quality of life. The measure has demonstrated face, construct and concurrent validity when compared with the Lancashire Quality of Life Profile [27].
8. Experiences of attending Accident & Emergency Questionnaire, devised for this study, assessed post-enrollment (see Appendix 2). The questionnaire had seven statements to assess the patients’ experience with the non-mental health ED staff and eight statements to assess the patients’ experience with the mental health professional in the ED. Scores ranged from 1 to 6, with higher scores reflecting better interactions with staff. There were two additional questions assessing whether patients were accompanied by family members/friends in the A&E and whether were they included in conversations with the mental health professionals (not enough, about the right amount or too much).
9. Service use data, using the Client Service Receipt Inventory (CSRI), which asks patients about all health care contacts over the past 6 months.

#### Qualitative interviews

At consent, patient and practitioner participants were given the option to take part in an interview about their experiences. All patients and practitioners who consented to this, in the intervention arm, were invited to take part in an interview towards the end of their involvement in the study. In total, 13 practitioners and 27 patients were interviewed. These were conducted face-to-face, over the phone or on Microsoft Teams by a research assistant. The research assistant facilitated the interview following a semi-structured interview schedule (Appendix 3), which explored practitioners’ and patients’ experiences of the intervention and taking part in the study. In this paper, we report on their experiences of taking part in the study. Their experiences of the intervention will be reported elsewhere as the focus of this paper is on the feasibility of the RCT.

#### Assessing fidelity to the intervention

Where practitioners and patients had consented to the option of their sessions being recorded, intervention sessions were audio-/video-recorded. A sample of these recordings was rated using a fidelity scale (Appendix 4), developed for this study, to assess the practitioners’ fidelity to the intervention manual. Sessions were rated by members of the research team on the fidelity scale. Eleven ED and 14 follow-up sessions were rated. Items for each component of the intervention (narrative interview, safety plan and solution focussed practice) were rated as ‘not done, ‘done to some extent’ or ‘done’.

### Data analysis

Descriptive statistics of patient and practitioner participant demographics and scores on all measures were calculated in Stata V17.0. Data were summarised descriptively for all participants and by the study arm. As this was a feasibility study, the statistical significance of any group differences was not tested.

Qualitative analysis of the interviews was conducted using framework analysis [28] to explore patients’ and practitioners’ experiences of taking part in the study. A separate analysis was undertaken for data relating to participants’ experiences of the intervention, which were reported elsewhere [29]. The data relating to experiences of taking part in the study were coded into a framework of three domains: motivations for taking part and experiences of the research procedures and sessions being recorded. These were explored to identify themes from the perspectives of patients and practitioners.

### Patient and public involvement

The LEAP was set up at the start of the project, which included seven members of the public with experience of presenting to the ED with self-harm or suicidality. They were involved in a number of aspects of the project, including identifying patient needs, refining the intervention, developing the protocol and measures (the self-harm survey and experiences of care in ED measure) and training practitioners in the intervention alongside the research team. The LEAP supported the project throughout phases 1 and 2.

### Ethical considerations

Ethical approval for phase 1 was obtained from the London-Surrey Borders Research Ethics Committee (Ref: 19/LO/0778). Practitioner and patient participants provided written consent for this study.

## Phase 2: Methods

### Design

The study was the internal pilot of a cluster randomised controlled trial to evaluate the clinical- and cost-effectiveness of the Assured intervention to patients presenting with self-harm and/or suicidal ideation. We aimed to recruit 10 ED sites to take part in the study. In practice, mental health practitioners from psychiatric liaison teams in 6 EDs were recruited. Practitioners were the clusters, who were consented and randomised to deliver the Assured intervention or TAU. The practitioners taking part in the study delivered the Assured approach or TAU to patients who met the eligibility criteria and consented to take part in the trial according to their assignment to the intervention or TAU arm. To mitigate against contamination, practitioners in the intervention arm were asked not to share details of practice with other colleagues in their team. It was not expected to be an issue given that the majority of the intervention was provided in follow-up sessions which the teams did not routinely provide.

### Feasibility outcomes

The feasibility outcomes for the internal pilot trial assessed whether the stop-go criteria for progression to the main trial were met at 9 months (a total of 14 months of the recruitment period). Stop-go criteria (see Table 1) assessed:

i. Recruitment: aim to recruit at least 70% of the recruitment target.
ii. Implementation of the intervention: aim for at least 70% of participants in the intervention arm to receive the ED assessment, safety plan and at least one follow-up session.
iii. Primary efficacy outcome data extraction: aim to obtain primary efficacy outcome data for at least 90% of participants.

**Table 1:** Stop/Go criteria to assess progression from the internal pilot (phase 2) to the main trial at month 9

	Green	Amber	Red
**Recruitment ** Target: recruit 702 participants by month 9 *i.e. 78 participants recruited per month, required to reach an overall sample size of* ***1088*** *by month 14*	>70% of recruitment target of 702 by Month 9 (*n* = 491)	40–69% of recruitment target of 702 by Month 9 (*n* = 485–281)	39% or less of the recruitment target of 702 by month 9 (*n* = 274)
**Intervention implementation** Based on the patient receiving ED assessment, safety plan and at least one follow-up session with:	>70% participants	50–69% participants	<49% participants
**Primary efficacy outcome** Outcome data can be extracted	For 90% participants	For 65–89% participants	For <65% participants
**ACTION**	Proceed to the main trial	Evaluate with PSC and funder	Likely that the trial would not proceed – decision evaluated with PSC and funder

*CI *Confidence interval, *PSC *Programme Steering Committee

This specified targets and indicators of serious problems. If any one of the criteria were amber, this required review with the Programme Steering Group (PSC) and the funder to identify whether we could implement changes to improve progress towards these criteria. If the criteria were red, it was considered unlikely that the full trial would go ahead.

### Participants

Practitioner and patient inclusion criteria were the same as described in phase 1.

Practitioners who consented to take part were randomised to a study arm. The allocation ratio was 1:1, and practitioners were randomised in block sizes of 2 and 4 using stratification by site. Those assigned to the Assured arm received 2 days of training in the intervention.

Patient allocation was based on the practitioner they were assigned to for their biopsychosocial assessment i.e. if their allocated practitioner had been trained in the Assured approach, they would receive the intervention and if their allocated practitioner had been assigned to deliver TAU they would receive TAU.

### Sample size

#### Sample size for a full trial

We aimed to recruit 92 practitioners and 1088 patients in total including those recruited in the internal pilot and the main trial. This was based on a power calculation with a binary primary efficacy outcome i.e. reattendance of patient with self-harm within 18 months (0 = no reattendance, 1 = at least one reattendance). This was based expected 30% reattendance rate in TAU [3]. This was estimated from the literature as we were unable to estimate the reattendance rate from the feasibility study as the follow-up period was 6 months due to funding constraints. Reducing reattendance from 30 to 20% over 18 months would reflect an important difference, corresponding to 22,000 fewer ED contacts. Based on a 30% reattendance rate in TAU and 20% rate in the intervention arm and ICC of 0.03 to capture clustering between participants assigned to the same clinician, a design based on uniform cluster size (i.e. 12 patients per clinician, figure approximate to what was anticipated) a sample size of 784 was required to achieve 90% power and type I error probability of 5%. In practice, some variation in cluster size was anticipated—on that basis, this preliminary figure was adjusted upward using an inflation factor of 1.386 based on a range of cluster sizes from *n* = 1 to *n* = 20 and a mean size of 12 [30]. This correction returned a final sample size estimate of 1088, assuming no loss to follow-up in the study population, a reasonable assumption given the use of routine data to measure the primary efficacy outcome and the findings from phase 1.

#### Sample size justification for internal pilot

The target sample size for the internal pilot was 491 patients (i.e. 70% of the expected 700/1088) to be recruited by month 9. Achieving 70% (3.5% margin of error with 95% confidence) of the month 9 target would indicate that we were on track to meet the overall target by month 14. Assuming 90% of primary outcome data can be extracted, with *n* = 491, this could be estimated to be within 2.8% with 95% confidence).

### Intervention and TAU

The Assured intervention and TAU are as described in phase 1.

### Procedures

#### Practitioner recruitment and data collection

Practitioners were approached about the study via the team manager. Written consent was obtained, and practitioners were then asked to complete a sociodemographic questionnaire and a validated measure of staff burnout measure: the MBI [17]. Practitioners were then randomised to a study arm by the trial manager using a Research Electronic Data Capture (REDCap) database. The practitioner and their team manager were then informed of the randomization outcome. For those randomised to deliver the Assured approach, their training in the intervention was then arranged.

#### Patient recruitment and data collection

Patients presenting to the ED with self-harm and/or suicidal ideation were recruited into the study. The study ran from July 2022 to March 2023. By this point, the COVID-19 pandemic had subsided, meaning that researchers were permitted to be on-site and all recruitment of patients could be done by an on-site researcher. This follows the first consent procedure outlined in the methods for phase 1.

The patient was referred to the liaison psychiatry team for a biopsychosocial assessment following a presentation to the ED for self-harm and/or suicidal ideation. After the referral to liaison psychiatry, the practitioner asked the patient if they were interested in speaking to a researcher about the study. With the patients’ agreement, the researcher provided the patient with the Participant Information Sheet (PIS) and obtained written consent. The researcher then left and the practitioner conducted the biopsychosocial assessment. Patients were not informed of their study arm allocation prior to consent.

The follow-up period was 18 months. The first research assessment was scheduled as soon as possible after the biopsychosocial assessment, ideally within 1 week. The subsequent research assessments were scheduled for 3, 9 and 18 months. Research assessments could take place in person or over a phone/video call.

#### Measures

The measures collected in the internal pilot are summarised in the supplementary appendices. As per phase 1, the primary efficacy outcome was repeat self-harm resulting in representation to the ED at 18 months, identified by searching ED electronic records, using the gold standard approach developed in the Multicentre Study of Self-Harm in England [21].

Some changes were made to the secondary outcomes from phase 1. Specifically:

1. Self-reported self-harm, using text message data collection at 1, 2, 3, 9 and 18 months (see Appendix 5). This was simplified from the version used in the feasibility study to enable it to be collected via text message, instead of an electronic survey sent by email survey. This method was changed with the aim of improving completion rates.
2. Suicidal ideation, using the Beck Scale for Suicide Ideation (BSS) in all four research assessments at the ED and at 3, 9, and 18 months [31]. This replaced the C-SSRS to provide a briefer measure of suicidality that could be collected by self-report. The BSS shows good internal consistency in outpatient samples [32] and convergent validity [33, 34].
3. Psychological wellbeing – Warwick-Edinburgh Mental Wellbeing Scales (WEMWBS) in all four research assessments at the ED and at 3, 9, and 18 months [35]. This was selected to replace the MANSA. The WEMWBS has been demonstrated to have strong internal consistency, construct validity and test-retest reliability [35].

### Data analysis

Descriptive statistics were calculated in Stata V17.0. Data are summarised descriptively against each criterion in the stop-go criteria.

### Ethical considerations

Ethical approval for phase 2 was obtained from London – City and East Research Ethics Committee (Ref: 21/LO/0683). Practitioner and patient participants provided written consent for this study.

## Phase 1: Results

The findings from phase 1 indicated that we could feasibly recruit patients presenting to the ED with self-harm and/or suicidal ideation to a study testing a brief psychological intervention, obtain patient and practitioner data, that the research methods were acceptable to patients, and that the intervention could be delivered with acceptable engagement and fidelity to the intervention manual. Thus, it was warranted to progress to the internal pilot trial. We report on the results from the internal pilot trial.

### Objective 1: Test the feasibility of recruiting patients from the ED into the study

We assessed whether we could recruit practitioners and patients into the study from four EDs. We successfully recruited 22 practitioners into the study, exceeding our target of 20 practitioners. Of these, six delivered TAU and 16 delivered the intervention and received 2 days of training in the intervention.

**Fig. 1:**
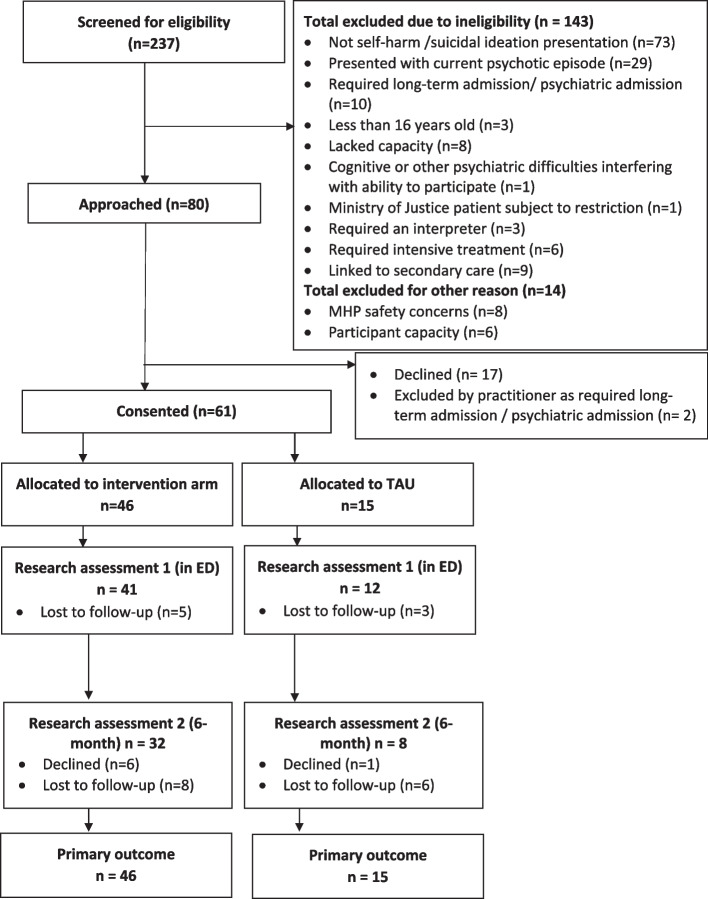
Consort diagram (participants). *MHP* Mental Health Practitioner

The patient flow diagram is shown in Fig. 1. The screening information is based on when researchers were on-site and could record this. This is therefore incomplete, as for most of the project, researchers were unable to be on-site due to the COVID-19 pandemic, and screening information could not be reliably obtained.

Of the 80 patients who were eligible and approached to take part, 19 were not consented (17 declined and 2 were excluded by the practitioner due to them requiring a long stay admission). We recruited 61 patients in total, 46 of whom were allocated to the intervention arm and 15 to TAU.

Demographic characteristics for patients are shown in Table 2. Approximately two thirds were female (73.9% in the intervention arm; 53.3% in TAU). Patients had a mean age of 30.2 and 29.0 in the intervention and TAU arms, respectively. The majority identified as White (76.9% in the intervention arm; 70% in TAU).

**Table 2:** Patient demographic data for phase 1

	Number with complete data, *n* (%)		Summary measure	
	Intervention	TAU	Intervention	TAU
**Gender - *n*(%)**	46 (100.0)	15 (100.0)		
Female			34 (73.9)	8 (53.3)
Male			12 (26.1)	7 (46.7)
Prefer not to say			0 (0.0)	0 (0.0)
Prefer to self-describe			0 (0.0)	0 (0.0)
**Age (years) – mean (SD) [ci]**	45 (97.8)	15 (100.0)	30.2 (11.9) [26.6-33.7]	29.0 (9.9) [23.5-34.5]
**Ethnicity –** ***n*(%)**	39 (84.8)	10 (66.7)		
White			30 (76.9)	7 (70.0)
Mixed			0 (0.0)	1 (10.0)
Asian			6 (15.4)	2 (20.0)
Black			2 (5.1)	0 (0.0)
Other			1 (2.6)	0 (0.0)
**Employment –** ***n*(%)**	39 (84.8)	11 (73.3)		
Paid or self-employment (full-time)			11 (28.2)	2 (18.2)
Paid or self-employment (part-time)			8 (20.5)	1 (9.1)
Voluntary employment (unpaid)			0 (0.0)	0 (0.0)
Sheltered employment			0 (0.0)	0 (0.0)
Unemployed			9 (23.1)	4 (36.4)
Student			8 (20.5)	4 (36.4)
Housewife/husband			0 (0.0)	0 (0.0)
Retired			2 (5.1)	0 (0.0)
Other			1 (2.6)	0 (0.0)
**Education –** ***n*(%)**	39 (84.8)	10 (66.7)		
Primary education or less			0 (0.0)	0 (0.0)
Secondary education			28 (71.8)	7 (70.0)
Tertiary/further education			11 (28.2)	3 (30.0)
Other general education			0 (0.0)	0 (0.0)
**Marital status –** ***n*(%)**	39 (84.8)	10 (66.7)		
Single/unmarried			28 (71.8)	9 (90.0)
Married/civil partnership			4 (10.3)	1 (10.0)
Co-habiting			5 (12.8)	0 (0.0)
Separated			1 (2.6)	0 (0.0)
Divorced			1 (2.6)	0 (0.0)
Widow/widower			0 (0.0)	0 (0.0)
**Accommodation type –** ***n*(%)**	39 (84.8)	11 (73.3)		
Independent			37 (94.9)	10 (90.9)
Supported			2 (5.1)	1 (9.1)
Homeless/roofless			0 (0.0)	0 (0.0)
Other			0 (0.0)	0 (0.0)
**Living situation –** ***n*(%)**	39 (84.8)	11 (73.3)		
Living alone			6 (15.4)	1 (9.1)
Living with a partner or family			25 (64.1)	5 (45.5)
Living with friend(s)			5 (12.8)	4 (36.4)
Living in shared accommodation			2 (5.1)	1 (9.1)
**Physical health conditions –** ***n*(%)**	39 (84.8)	10 (66.7)		
Yes			17 (43.6)	5 (50.0)
No			22 (56.4)	5 (50.0)
**Current psychiatric diagnosis –** ***n*(%)**	39 (84.8)	10 (66.7)		
Yes			21 (53.8)	7 (70.0)
No			18 (46.2)	3 (30.0)
**Previous admission to psychiatric hospital –** ***n*(%)**	39 (84.8)	11 (73.3)		
Yes			9 (23.1)	3 (27.3)
No			30 (76.9)	8 (72.7)

*INT *Intervention, *TAU* Treatment as usual, *CI* Confidence intervention, *SD* Standard deviation

### Objective 2: Pilot outcome measures in the ED setting for a future trial

#### Research assessments 1 and 2

Secondary outcome measures were obtained in research assessments—the first of which was in the days following the index ED presentation and the second of which was 6 months later. Research assessment 1 was completed for 87% of patients, with the rest being unavailable or unable to be contacted. Research assessment 2 was completed at 6 months by 66% of patients. To assess patient-rated outcome measures in the ED setting, each outcome is summarised by treatment group at post-ED assessment and at 6 months (Table 3). We report on the scores on each measure below but emphasise caution in interpreting these findings due to the small sample size.

**Table 3:** Patient outcomes at research assessments 1 and 2 in phase 1

	Research assessment 1				Research assessment 2			
	Number with complete data *n* (%)		Summary measure		Number with complete data *n* (%)		Summary measure	
Outcome measure	INT	TAU	INT	TAU	INT	TAU	INT	TAU
**CSSRS**								
Suicidal Ideation—*n*(%)	38 (82.3)	10 (66.7)			29 (63.0)	7 (46.7)		
Present			37 (97.4)	8 (80.0)			15 (51.7)	4 (57.1)
Not present			1 (2.6)	2 (20.0)			14 (48.3)	3 (42.9)
Intensity of ideation—mean (sd) [ci]	35 (76.1)	8 (53.3)	16.8 (5.2) [15.0–18.5]	14.8 (4.1) [11.3–18.2]	13 (28.3)	4 (26.7)	13.4 (3.9) [11.0–15.8]	11.5 (4.7) [4.1–18.9]
Suicidal behaviour—*n*(%)	37 (80.4)	10 (66.7)			28 (60.9)	7 (46.7)		
Present			32 (86.5)	7 (70.0)			3 (10.7)	1 (14.3)
Not present			5 (13.5)	3 (30.0)			25 (89.3)	6 (85.7)
**CORE-OM—mean (sd)[ci]**	35 (76.1)	11 (73.3)	2.5 (0.7) [2.3–2.8]	2.5 (0.5) [2.1–2.8]	26 (56.5)	6 (40.0)	1.8 (1.0) [1.4–2.2]	1.3 (0.5) [0.8–1.8]
**SIX—mean(sd)[ci]**	19 (41.3)	11 (73.3)	1.9 (0.5) [1.6–2.1]	1.9 (0.6) [1.5–2.3]	28 (60.9)	7 (46.7)	1.8 (0.6) [1.6–2.0]	1.9 (0.6) [1.4–2.4]
**MANSA—mean(sd)[ci]**	21 (45.7)	10 (66.7)	3.6 (1.0) [3.2–4.1]	3.6 (0.8) [3.0–4.2]	22 (47.8)	6 (40.0)	4.3 (1.2) [3.8–4.8]	4.6 (0.6) [4.0–5.2]
**Helping Alliance Scale—mean (SD) unless specified**								
Was the care you received right for you?	21 (45.7)	10 (66.7)	7.3 (2.2)	7.1 (1.4)	23 (50.0)	6 (40.0)	7.3 (2.4)	8.2 (1.6)
Did you feel understood by the practitioner?	21 (45.7)	10 (66.7)	7.1 (2.4)	7.9 (1.2)	22 (47.8)	6 (40.0)	7.6 (2.7)	8.7 (1.5)
Did you feel criticised by the practitioner?	21 (45.7)	10 (66.7)	2.0 (3.2)	0.6 (1.9)	23 (50.0)	6 (40.0)	1.3 (2.2)	1.2 (2.4)
Did you feel supported by the practitioner?	21 (45.7)	10 (66.7)	7.7 (1.8)	8.2 (1.3)	23 (50.0)	6 (40.0)	7.7 (2.6)	8.0 (2.3)
Did you trust in the practitioner and in his/her professional competence?	20 (43.5)	9 (60.0)	7.8 (2.4)	8.2 (1.4)	23 (50.0)	6 (40.0)	7.9 (2.7)	8.8 (1.3)
How did you feel immediately after meeting with the practitioner? *n*(%)	21 (45.7)	10 (66.7)			23 (50.0)	6 (40.0)		
Worse			1 (4.8)	0 (0.0)			0 (0.0)	0 (0.0)
Unchanged			9 (42.9)	4 (40.0)			10 (43.5)	2 (33.3)
Better			11 (52.4)	6 (60.0)			13 (56.5)	4 (66.7)
**Experience in A&E—mean(sd) unless specified**								
With the general staff	21 (45.7)	8 (53.3)	3.3 (0.6)	3.5 (0.5)				
With the mental health professional	18 (39.1)	10 (66.7)	3.4 (0.8)	3.3 (0.7)				
Accompanied by family member/friend, *n* (%)	0 (0.0)	4 (26.7)						
Yes			0 (.)	1 (25.0)				
No			0 (.)	3 (75.0)				
If yes, were they included in the conversation with the mental health professional? *n* (%)	0 (0.0)	1 (6.7)						
Not enough			0 (.)	0 (0.0)				
About the right amount			0 (.)	1 (100.0)				
Too much			0 (.)	0 (0.0)				

*INT *Intervention, *TAU* Treatment as usual, *CSSRS* Columbia Suicide Severity Rating Scale, *CORE-OM* Clinical Outcomes in Routine Evaluation–Outcome Measure, *SIX* Social Outcomes Index, *MANSA* Manchester Short Assessment of Quality of Life, *CI* Confidence intervention, *SD* Standard deviation

Based on the Columbia-Suicide Severity Rating Scale (C-SSRS) at Research Assessment 1, we found that suicidal ideation was present in the past month in 97.4% of patients in the intervention arm and 80% of patients in TAU. Ratings of intensity on a scale of 2–25 showed similar levels of intensity in the intervention (*M* = 16.8) and TAU arms (*M* = 14.8). Suicidal behaviour was present in the past month in 86.5% of patients in the intervention arm and 70% of the patients in the TAU arm in research assessment 1. At research assessment 2, rates of suicidal ideation in the past month were lower than in research assessment 1 in both the intervention (51.7%) and TAU arm (57.1%), compared with research assessment 1. There was a reduction in suicidal ideation intensity in the intervention (*M* = 13.4) and TAU arm (*M* = 11.5). There was also a reduction in suicidal behaviour in both arms by research assessment 2, with suicidal behaviour present in the past month in 10.7% of participants in the intervention arm and 14.3% of participants in the TAU arm. Ratings on the C-SSRS are shown in Table 3.

Average scores on the CORE-OM were 2.5 in both the intervention and TAU arm at research assessment 1. In research assessment 2, they were 1.8 and 1.3 in the intervention and TAU arms, respectively, reflecting an improvement in functioning. Social outcomes, measured with the SIX, remained unchanged in both the intervention and TAU arms at 6 months. Quality of life improved in both study arms as reflected by an increase in scores on the MANSA, from 3.6 to 4.3 in the intervention arm and 3.6 to 4.6 in the TAU arm over the 6-month follow-up period.

Patients completed a measure of therapeutic alliance, to examine the relationship between patients and the practitioner they saw. Findings show that in both the intervention and TAU arms, scores showed a good therapeutic alliance as reported by patients with regard to the practitioner they saw. The majority of patients reported feeling better after meeting with the practitioner. Some reported feeling unchanged after meeting with the practitioner, and only one patient in the intervention arm reported feeling worse after meeting with the practitioner.

The final self-report measure sought to capture patients’ experiences of the care they received in the ED (Table 3). Items asked patients to rate statements on a scale of 1 to 6 in relation to interactions with generalist ED staff and the specialist mental health practitioner, with higher scores reflecting better interactions with staff. Average ratings were in the middle of the range, generally reflecting acceptable interactions with staff but with room for improvement.

#### Monthly self-report self-harm

Patients received a monthly email survey to capture self-report self-harm presentations over the past month (Table 4). This survey was subject to a substantial level of missing data. Of the 61 patients, only 21 completed the survey at least once. Completion rates in the intervention arm ranged between 15.2 and 23.9% at each timepoint and in TAU between 6.7 and 20.0%. This highlights issues in the feasibility of obtaining self-report self-harm data via an email survey. The data collected is reported in Table 4.

**Table 4:** Self-report self-harm data collected with an online survey, at months 1–6, for phase 1

	Intervention (*n* = 46)		TAU (*n* = 15)	
Month	Self-harm survey complete *n* (%)	Of those, self-harm reported *n* (%)	Self-harm survey complete *n* (%)	Of those, self-harm reported *n* (%)
**1 **	7 (15.2%)	3 (42.9%)	1 (6.7%)	1 (100%)
**2 **	11 (23.9%)	3 (27.3%)	3 (20.0%)	1 (33%)
**3 **	10 (21.7%)	4 (40.0%)	3 (20.0%)	1 (33%)
**4 **	8 (17.4%)	4 (50.0%)	3 (20.0%)	0 (0%)
**5 **	8 (17.4%)	3 (37.5%)	3 (20.0%)	0 (0%)
**6 **	9 (19.6%)	4 (44.4%)	3 (20.0%)	1 (33%)

Responses to online survey which asked participants whether they had self-harmed in the past month

#### Data extraction from hospital electronic records

The proposed primary efficacy outcome for the future RCT was repeat self-harm presentations to the ED over the 6-month follow-up period. These outcome data were obtained for the entire sample demonstrating the feasibility of extracting primary efficacy outcome data from medical electronic records for patients. Findings show a higher rate of re-attendance to the hospital for self-harm over 6 months in the TAU arm (20%) compared with the intervention arm (13%) in this sample and that no patients died by suspected suicide over the 6-month follow-up period (Table 5).

**Table 5:** Outcome data extracted from hospital electronic records for phase 1

	Number with complete data (%)		Summary measure	
	Intervention	TAU	Intervention	TAU
**Repeat hospital presentation for self-harm—*n*** **(%)**	46 (100.0)	15 (100.0)		
Yes			5 (10.9)	3 (20.0)
No			41 (89.1)	12 (80.0)
**Death by suicide—*n*** **(%)**	46 (100.0)	15 (100.0)		
Yes			0 (0.0)	0 (0.0)
No			46 (100.0)	15 (100.0)

Overall, our findings show that it was feasible to obtain outcome data from electronic records. We were able to obtain self-report measures for patients, when administered by researchers, although subject to a degree of loss to follow-up throughout the follow-up period. However, the self-report self-harm survey, administered by an automatic email, had substantial missing data.

### Objective 3: Pilot collection of practitioner-report data

Practitioner-reported data were collected at the start of the study, to obtain practitioner sociodemographic information, professional role/history and burnout, using the MBI (see Table 6). The majority of practitioners were female (intervention arm = 70.6%; TAU arm = 83.3), and the majority were nurses (intervention arm = 70.6%; TAU arm = 83.3%). They had an average age of 43.7 and 35.0 in the intervention and TAU arms, respectively. Practitioners in the intervention arm on average had more years’ of experience working in mental health services (*M* = 15.3) compared with practitioners in TAU (*M* = 10.3). This may reflect the self-selection of the study arms in the feasibility study or may be down to chance given the small sample.

**Table 6:** Practitioner data for phase 1

	Number with complete data *n* (%)		Summary measure	
	Intervention	TAU	Intervention	TAU
**Gender—*n*** **(%)**	17 (94.4)	6 (100.0)		
Female			12 (70.6)	5 (83.3)
Male			5 (29.4)	1 (16.7)
Non-binary			0 (0.0)	0 (0.0)
Prefer not to say			0 (0.0)	0 (0.0)
**Age (years)—mean (sd) [ci]**	10 (55.6)	3 (50.0)	43.7 (6.2) [39.3–48.1]	35.0 (8.9) [12.9–57.1]
**Ethnicity—*n*(%)**	10 (55.6)	3 (50.0)		
White			4 (40.0)	2 (66.7)
Mixed			0 (0.0)	0 (0.0)
Asian			0 (0.0)	1 (33.3)
Black			5 (50.0)	0 (0.0)
Chinese			1 (10.0)	0 (0.0)
Other			0 (0.0)	0 (0.0)
**First language—*n*(%)**	10 (55.6)	3 (50.0)		
English			5 (50.0)	3 (100.0)
Other			5 (50.0)	0 (0.0)
**Professional role—*n*(%)**	17 (94.4)	6 (100.0)		
Nurse			12 (70.6)	5 (83.3)
Doctor			2 (11.8)	1 (16.7)
Social worker			0 (0.0)	0 (0.0)
Support worker			1 (5.9)	0 (0.0)
Psychologist			2 (11.8)	0 (0.0)
**Experience in liaison psychiatry** **(years)—mean (SD) [ci]**	10 (55.6)	3 (50.0)	6.0 (4.1) [3.0-9.0]	1.3 (1.2) [−1.5–4.2]
**Experience in mental health services** **(years)—mean (sd) [ci]**	10 (55.6)	3 (50.0)	15.3 (8.0) [9.6–21.0]	10.3 (11.0) [−16.9–37.6]
**Previous training in self-harm and/or suicide—*n*(%)**	10 (55.6)	3 (50.0)		
Yes			6 (60.0)	2 (66.7)
No			4 (40.0)	1 (33.3)
**Maslach Burnout Inventory—mean (sd) [ci]**				
Prior to allocation				
Emotional exhaustion	9 (50.0)	3 (50.0)	15.3 (5.0) [11.5–19.2]	17.0 (5.6) [3.2–30.8]
Personal achievement	9 (50.0)	3 (50.0)	46.6 (7.8) [40.6–52.5]	45.0 (6.1) [29.9–60.1]
Depersonalization	9 (50.0)	3 (50.0)	6.0 (1.7) [4.7–7.3]	7.3 (0.6) [5.9–8.8]

*INT* Intervention, *TAU* Treatment as usual, *CI* Confidence intervention, *SD* Standard deviation

Practitioners were asked to complete the MBI at the start of their involvement in the study. Average scores were similar for intervention and TAU practitioners.

Our findings show that it was feasible to obtain self-report questionnaire data from practitioners.

### Objective 4: To explore patients’ and practitioners’ experiences of taking part in the study

Analysis of interviews revealed that the research procedures were generally acceptable to practitioner and patient participants, who took part in the study with a wish to improve care for others in the future.

#### Motivated to take part to help other people

Patients spoke positively about being invited to take part in the study, as it made them feel that they were not the only person going through a mental health crisis and it made them feel there are people trying to find a solution to these issues. Patients spoke about their motivation to take part, which centred entirely around a wish to help other people experiencing similar difficulties. Patients expressed an understanding that while taking part may not directly benefit them, hoped that it would help others:

It’s not easy to talk about these things but if it's going to help even just one person to not go through all of this then I’m happy. I’m happy I’ve done it. And overall it was a very positive experience. (Patient)

This reflects that participation was for altruistic reasons, with patients hoping the difficulties they had experienced may improve care for others in the future.

#### Researchers were sensitive and had a personal touch

The researcher’s approach was important through all aspects of patients’ involvement in the study. Patients spoke positively about interactions with researchers, who felt supported them to make the decision on whether to take part in the study:

She went about it in like a really professional and nice way. And I didn't feel pressurized, that I had to do it, and I genuinely, genuinely felt like it was my choice. (Patient)

Patients spoke about how to contact from researchers was personalised, such as thank you notes and having options around how they were contacted (e.g. email or text message). This was received positively by patients:

It’s personal, it was nice, nothing felt erm like too much hard work. It wasn’t like a typed letter and stuff like that. As I said to you like, the little things you do, the texting to let me know this was ready or that was ready or I was going to phone you or you know that, all of that, your personal touch helped. (Patient)

When completing questionnaires with a researcher, including about difficult topics such as self-harm and suicidality, a sensitive approach from researchers helped mitigate distress. For example, one patient said:

And I like the fact that you’ve kind of warned me that some of them are a bit tough and might be hard to answer kind of thing not just go straight in with questions that like trigger or something like that. (Patient)

This approach was important on the scales asking sensitive topics, such as the C-SSRS. Similarly, another patient said:

Some of them were quite difficult questions but he was very good at sort of like, helping me answer them as well and knowing which bits to follow up on and ask if I needed more detail with or suggestions if I didn’t know where to start with the answer. And he was really good at saying like, ‘are you alright?’ or ‘would you like to take a break?’, ‘do you need a few minutes?’. And I really like how both of them checked in on how I was at the end. Like, ‘are you alright after answering all these questions?’. (Patient)

Overall, a sensitive approach from researchers was crucial for patients, who described researchers as understanding and empathetic.

Similarly, practitioners spoke positively about interactions with researchers. However, one aspect of the research that practitioners suggested could be improved was the timing of when the study was first introduced to patients, referring to those where the patient was consented prior to their biopsychosocial assessment in the ED. They suggested it would be better for patients to be contacted about the study after leaving the ED (as was done in some cases):

And then in that time, I think, they are not in crisis, they are not in A&E… then they can think ‘do I really want to take part in this or not?’. Because when they are in A&E, they are in crisis, they will accept anything if they think it’s gonna be helpful. (Practitioner)

This reflects the challenges of recruiting patients to a study in the ED setting, and how to offer research to patients while acknowledging the distress they are experiencing and the time constraints owing both to the urgent care required and the targets for discharge from the ED (i.e. the requirement for patients to be assessed within 1 h of referral to liaison psychiatry and to be discharged within 4 h of presenting to the ED).

#### Research questionnaires could be overwhelming and restrictive

Patients described how the questionnaires could be overwhelming for them, particularly if they were contacted when they were having a difficult time:

Yeah it’s been fine as long as I feel a clear head the day that I’ve had these calls or have to do the questionnaires anyway. I know if I’ve had like a really rough day I normally leave them a day or two. But yeah, it’s been fine. (Patient)

Patients needed to feel in a good enough place to complete the research questionnaires, as it could otherwise have been triggering for them:

If I was having a really bad day and I had some of those same questions then I probably would have like just been upset about it and probably would have affected me but I wasn’t having a really bad day so um I was a little bit triggered but I was fine I was able to deal with it you know? (Patient)

Some patients spoke about completing the questionnaires in more than one phone call or meeting with the researcher, to make it feel less overwhelming for them.

Other patients spoke about the limitations of the questionnaires, describing how they sometimes missed the context of what was going on in their lives. Some spoke about the scales on the questionnaires and issues they experienced with these:

It was erm, very close between certain [items] and if anything, it was just trying to erm, yeah, I think if I were given a, erm not a multiple choice answer then I think it would’ve been a different erm, yeah. (Patient)

The responses on the questionnaires for some were perceived as not always fitting with how they were thinking or feeling:

Like, there were some of them that I was trying to give explanations for but really you wasn’t looking for that explanation, you were looking for erm, one of those erm selected choices. But I think that some of them were very difficult to answer because erm, I felt that I needed to explain why, the contents of that question and not just the answer because I feel that it didn’t quite match to what I was, I was feeling or thinking. (Patient)

This reflects the limitations of the questionnaires as experienced by some patients, particularly those with multiple-choice answers such as the CORE-OM and the MANSA. In contrast, other patients did not report issues with the questionnaires.

#### Research questionnaires were acceptable and for some offered space for reflection on the progress they had made

Most patients gave the impression that taking part in the trial was acceptable and the questionnaires were easy to answer:

It’s relevant, I don’t think there’s anything err, that shouldn’t be there, or take, taken out, I think they’re very relevant. (Patient)

The questionnaires for most patients were perceived as relevant to the issues they were experiencing. While some patients felt restricted by the scale responses to questions, others found these easier to answer:

I was quite like, pleasantly surprised at how just like factual they were, it was like, yeah, more factual than how are you feeling about this? How does this make you feel? Because those are the type of questions, I find can be quite difficult and triggering. Whereas, I am quite good at doing, like, numbers. If that makes sense. (Patient)

In addition to most patients finding the questionnaires acceptable, some reported finding them helpful. Some described how the questionnaires provided them with space to reflect on the progress they had made and to understand themselves better:

It’s good, it’s good and it also gave me an opportunity to understand my life, like how the steps were and where I was before and where I am. (Patient)

Similarly, another patient said:

I feel like it’s good to reflect on things. So it didn’t feel too - I remember when I signed up for it, I was worried that it would be a bit invasive. You know, like you speak to your therapist and then get asked ‘oh, what did you speak to your therapist about?’. But it hasn’t really felt like that. It’s been quite good to reflect and stuff. And it has felt like it’s more of a check-in as well – it doesn’t feel like you’re just firing questions at me. You know, it’s also a check-in on how I’m doing and stuff, it’s nice to look at my progress. (Patient)

The findings suggest the questionnaires were, on the whole, acceptable for patients.

#### Recording sessions were acceptable for patients, but practitioners could feel scrutinised

Practitioners and patients were asked how they felt about their intervention sessions being recorded. All patients who were interviewed were indifferent about being recorded, such as one patient who said they had ‘no particular feelings about it really’ and others who said ‘it doesn’t bother me’ and ‘it’s absolutely fine’. Several patients spoke about forgetting the sessions were being recorded once the session began. Practitioners on the other hand often expressed anxiety about being recorded, as it made them feel conscious of how they were delivering the session:

The thought of being recorded did make me very conscious of what I’m actually saying (Practitioner)

Similarly, practitioners spoke about concerns about being recorded delivering the intervention because of not feeling confident in it:

Well, [being recorded], it’s not my favourite thing to be recorded doing anything frankly, so you know, particularly doing something that you’re probably not very good at [laughs] (Practitioner)

Practitioners were concerned about being evaluated, whereas patients did not express concerns about being recorded.

### Objective 5: To explore patients’ engagement in the intervention

Of the 46 patients in the intervention arm, 28 (61%) attended at least one of the three follow-up sessions. Twenty patients attended all three sessions (44%), two patients attended two sessions (4%), six patients attended one session (13%) and 18 patients attended none of the offered sessions (39%). This shows that a majority of patients attended either no sessions or all three of the offered follow-up sessions.

### Objective 6: To explore practitioner fidelity to the intervention manual

Eighty-three percent of participants consented to their sessions being audio-recorded, and 82% consented to their sessions being video-recorded. Despite the high consent rate, it was often not possible to record the sessions, as researchers were not on-site to provide technical support with the recording of sessions. Eleven ED sessions were recorded, all of which were rated for fidelity. Thirty-six follow-up sessions were recorded. A sub-sample was selected of 14 sessions which allowed variability in patients and practitioners.

Observer-rated fidelity ratings are shown in Table 7. The fidelity ratings show that for most ED psychosocial assessments, practitioners used the narrative interview techniques i.e. inviting the patient to tell their story, encouraging the patient to tell their story, validating the patients’ distress and normalising their experience. Negative practices (shutting the patient’s story down and questioning or disputing the patients’ story) were used infrequently. The third negative practice, introducing solutions to problems in the narrative interview, was used in 63.6% of sessions, reflecting practitioners sometimes began problem-solving earlier in the ED session which is not consistent with narrative interview techniques.

**Table 7:** Fidelity ratings for phase 1

	Rating *n* (%)		
	Not done 0 *n* (%)	To some extent 1 *n* (%)	Done 2 *n* (%)
**ED session: Narrative interview **			
Used narrative interview opening	1 (9.1%)	N/A	10 (90.9%)
Used narrative interview techniques to encourage the patient to tell their story	0 (0%)	2 (18.2%)	9 (81.8%)
Shuts the patient’s story down^a^	2(18.2%)	2(18.2%)	7 (63.6%)
Validates the patient’s distress	0 (0%)	2(18.2%)	9 (81.8%)
Start talking about solutions to problems^a^	4 (36.4%)	N/A	7 (63.6%)
Questions or dispute the patient’s story^a^	1 (9.1%)	N/A	10 (90.9%)
Normalises the patient’s experience	2 (18%)	N/A	9 (82%)
**ED session: Safety plan**			
Introduced the safety plan and its purpose	1 (9.1%)	N/A	10 (90.0%)
Asked about warning signs	0 (0%)	N/A	11 (100%)
Asked about distractions	3 (27.3%)	N/A	8 (72.7%)
Asked about changing environment	1 (9.1%)	6 (54.5%)	4 (36.4%)
Asked about people they trust	0 (0%)	5 (45.5%)	6 (54.5%)
Asked about professionals	2 (18.2%)	3 (27.3%)	6 (54.4%)
Identifies someone to share the safety plan with	4 (36.4%)	3 (27.3%)	4 (36.4%)
**Follow-up sessions: Solution-focussed practice **			
Encourages exploration of the patient’s ‘best hopes’	8 (57.1%)	2 (14.3%)	4 (28.6%)
Explores how they will notice future/further signs of progress	0 (0%)	2 (14.3%)	12 (85.7%)
Encourages exploration what’s already working or exploration of change	9 (64.3%)	0 (0%)	5 (35.7%)
Reviews safety plan	8 (57.1)	3 (21.4%)	3 (21.4%)
Explores resources that may be helpful for the patient	5 (35.7%)	1 (7.1%)	8 (57.1%)

^a^Items reverse scored; scores based on observer rating of audio/video recorded sessions

Regarding the safety plan in the ED psychosocial assessment, practitioners scored highly for introducing the safety plan, possibly aided by having a paper safety plan to work through in the session. Sessions were assessed on fidelity to asking about warning signs and four coping strategies (distractions, changing environment, people they trust and professionals). In most sessions, practitioners asked about warning signs. For the coping strategies, fidelity to the intervention was mixed. Most sessions were rated as these coping strategies having been done to at least some extent. Practitioners scored highest for asking about distractions (done in 72.7% of sessions), people they trust (54.5%), professionals (54.5%) and changing environment (36.4%). A number of these coping strategies were only done ‘to some extent’, reflecting practitioners often covered these components of the safety plan, but they did not explore barriers to the patient using these coping strategies or steps they could take to overcome barriers. The final step in the safety plan was to identify someone to share the safety plan with, which was not done in 36.4% of rated sessions.

For the solution-focussed follow-up sessions, ratings showed variation in fidelity to the intervention. Exploration of best hopes was not done in the majority (57.1%) of sessions and encouraging exploration of what’s already working was not done in the majority of sessions (64.3%). Exploring how the patient would notice further signs of progress was done, at least to some extent, in all sessions. The safety plan review was not undertaken in most sessions (57.1%), but practitioners more frequently explored resources that may be helpful for the patient, rated as ‘done’ in 57.1% of sessions and ‘to some extent’ in 7.1% of sessions.

Overall, these findings demonstrate reasonable fidelity to the intervention manual, but with areas where fidelity could be improved.

## Summary of Phase 1 results

The findings from phase 1 indicated that we could feasibly recruit patients presenting to the ED with selfharm and/or suicidal ideation to a study testing a brief psychological intervention, obtain patient and practitioner data, that the research methods were acceptable to patients, and that the intervention could be delivered with acceptable engagement and fidelity to the intervention manual. Thus, it was warranted to progress to the internal pilot trial. We report on the results from the internal pilot trial.

## Phase 2: Results

The internal pilot opened in July 2022 and ran for 9 months until March 2023.

During these 9 months, we opened six sites. We did not open all 10 sites during this time due to sites declining to take part or those that had previously agreed to take part withdrawing. This was because of sites being overstretched and understaffed, and concerns about practitioner capacity to deliver follow-ups. This situation had worsened post-COVID, with significant issues with staff turnover, burnout and sickness across a number of the proposed sites. There were also delays in getting approval from the Trusts.

**Fig. 2:**
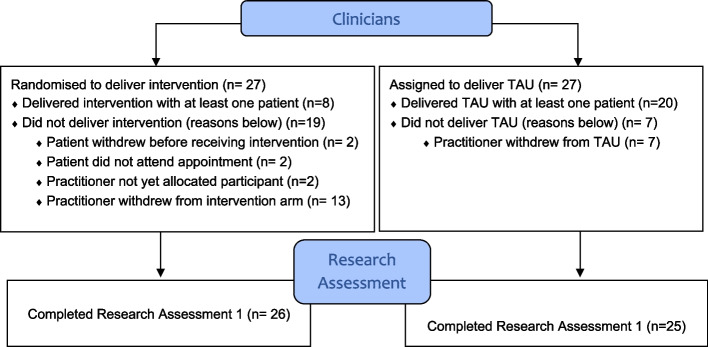
Consort diagram for clinicians for the internal pilot

From these six sites, we recruited and randomised 54 practitioners (see Fig. 2), towards our target of 92 practitioners. However, practitioner withdrawal became a significant issue. Of the 54 practitioners randomised, 20 withdrew. This was particularly a problem in the intervention arm, where 13 of the 20 withdrawn practitioners were in the intervention arm. Reasons for this were ‘did not agree with intervention approach’ (*n* = 2), ‘additional workload of follow-up’ (*n* = 2), ‘left the liaison team’ (*n* = 5) and ‘sickness/personal reasons’ (*n* = 4). In TAU, 7 of the 27 practitioners withdrew from the study as they left the team. While some practitioner turnover was anticipated due to staff leaving the team, we had not anticipated the high levels of practitioner withdrawal in the intervention arm.

In turn, this caused issues with patient recruitment. Ongoing efforts were made to recruit additional practitioners in all teams, but this was challenging due to practitioners declining to take part due to concerns about extra workload and delays in training practitioners due to team capacity.

We report on progress made towards our stop-go criteria below.

### Stop-go criterion 1: Recruitment

**Fig. 3:**
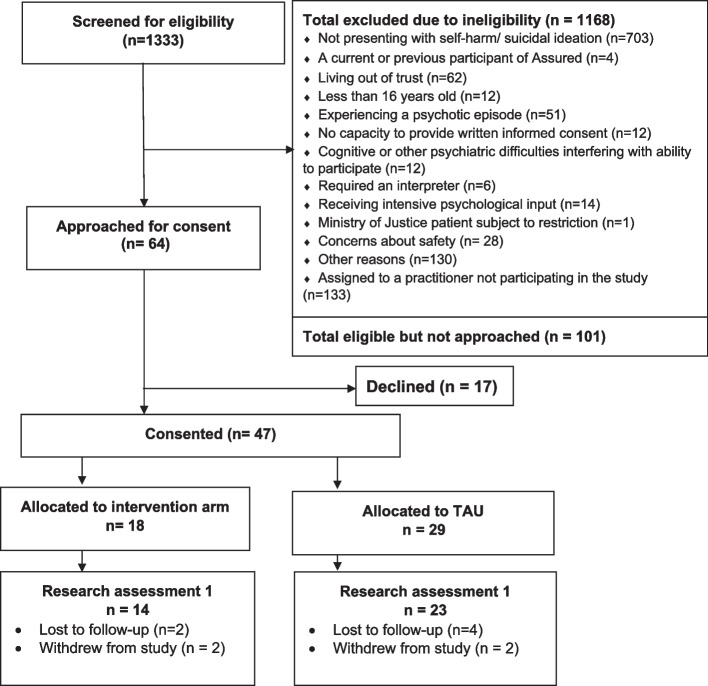
Patient consort for phase 2 internal pilot trial

The first stop-go criterion was recruitment, where our target was to recruit 78 patients per month. Over the 9 months, we recruited 47 patients in total, thus falling significantly short of our required recruitment of 491 as defined in the stop-go criteria (see Fig. 3).

Challenges in recruiting patients were for several reasons. To recruit a patient, an eligible patient needed to present to the ED meeting the inclusion criteria when a researcher was on-site to consent the patient and a practitioner taking part in the study was on shift and available to conduct the biopsychosocial assessment with the patient, within the required 1-h target from referral from the ED to the liaison psychiatry team. This proved to be problematic due to many eligible patients presenting out of hours when researchers tended not to be on site. Liaison practitioners also often worked nights, but researchers did not, meaning there were many shifts when it was not possible to recruit patients. Moreover, as we had fewer participating practitioners than planned, this reduced the hours in which we were able to recruit. Even for those practitioners who were taking part, they would often be unavailable at short notice due to being called to other duties to manage staff shortages. The recruitment period coincided with changes to the flow of patients in the ED, as there was a national bed crisis. This led to patients staying longer in ED and staff from liaison psychiatry teams being diverted from assessment work to managing beds impacting on practitioner availability. This led to the trial being stopped in March 2023, with a view to re-designing the trial based on our learning so far.

### Stop-go criterion 2: Implementation of the intervention

We assessed the implementation of the intervention, defined as the patient receiving the ED assessment, safety plan and at least one follow-up session. Of the 18 patients allocated to the intervention arm, the intervention was implemented for 12 patients (66%) i.e. amber according to this stop-go criterion. For those who did not receive the intervention, this was because the patient withdrew before the follow-up session was arranged (*n* = 2), the patient did not attend any sessions (*n* = 3) or the practitioner was not available for the sessions (*n* = 1).

### Stop-go criterion 3: Primary efficacy outcome data extraction

It was too early to obtain primary efficacy outcome data for participants, as the study endpoint was 18 months. Therefore, we were not in a position to assess this stop-go criterion, although from the feasibility study, this is not anticipated to be an issue as we successfully obtained these data for the entire sample at 6 months.

## Discussion

This is the first study in the UK to explore the feasibility of testing a brief psychological intervention in the ED, starting with a therapeutic psychosocial assessment in the ED and then rapid follow-up care. The enhanced psychosocial assessment in the ED included a narrative interview and personalised safety plan. Rapid follow-up care consisted of three sessions following a solution-focussed approach at around 1, 4 and 8 weeks. The feasibility study, with an adapted protocol during the pandemic, indicated that the methods were feasible and acceptable to both practitioners and patients. Based on these findings, we progressed to the internal pilot trial of a cluster randomised controlled trial. However, we did not meet our recruitment stop-go criteria—largely due to the need for researchers and practitioners to be on site together, many self-harm presentations occurring out of hours and the need to take consent from patients in the ED before they received a biopsychosocial assessment. Based on this, it was determined that the study should not progress to the main trial without substantial changes to the protocol.

The COVID-19 context in which the feasibility study took place means there were two pathways to recruitment. The first of which a researcher was on-site to consent patients prior to their biopsychosocial assessment. This is the approach we intend to use in the future RCT. However, due to COVID-19 restrictions, researchers were unable to be on-site for most of the recruitment period. Therefore, for many patients, consent was obtained remotely after the patient had left the ED. This meant that researchers and patients were unblinded to their allocation at the point of consent. When we progressed to the internal pilot trial, this approach was not possible, as if patients were aware of their allocation prior to consent this may influence their decision to take part, thus biasing the groups in each study arm. In the internal pilot trial, we consented patients prior to their biopsychosocial assessment, meaning that a researcher needed to be on-site when the patient was referred to the liaison psychiatry team and a practitioner taking part in the study needed to be available to pick up the referral. This proved not to be feasible in terms of achieving the desired recruitment rate.

Despite the challenges in recruiting in the internal pilot, one promising finding was the consent rate of 76% in phase 1 and 73% in phase 2, indicating that despite the challenges of recruiting patients in distress, they were often agreeable to taking part. This figure is similar to the 70% consent rate in the recent safety planning study in Scotland, SAFETEL, where patients were also recruited in EDs [36]. This reflects the feasibility of consenting patients, even in the ED context. Qualitative findings from phase 1 indicated that patients were motivated to take part in this research as they had a desire to help improve care for others in the future, as found in previous studies [37]. These findings reflect that the barrier to recruitment was due to the ED logistics and time-limited window in which we had to approach patients for consent, rather than patients not wishing to take part.

Other findings from this study suggest that a trial of the Assured intervention is warranted with adaptation to the recruitment methods. In phase 1, we successfully recruited a diverse sample, in terms of ethnicity and employment status. The intervention was delivered with reasonable fidelity to the manual. While there were areas that could be improved with regard to fidelity, we anticipate that increased training and supervision for practitioners would support improved fidelity. In phase 1, 61% of patients in the intervention arm attended at least one follow-up session, highlighting that when offered follow-up support, the majority took this up. In the immediate aftermath following a self-harm presentation to the hospital, patients are at increased risk of suicide [38], so from a suicide prevention perspective this has the potential to provide a lifeline at a time when people are most at risk. This is particularly important given the barriers to accessing psychological treatment for self-harm [39]. In a subsequent study, we explored the experiences of the Assured intervention for practitioners and patients who participated in phase 1. Findings indicated that the Assured intervention gives agency and hope to highly distressed patients, provides validation of their emotions and supports with their mental health. The intervention was found to re-imagine the practitioner-patient relationship, facilitating trust for patients who have often had difficult help-seeking histories, which was rewarding for practitioners, to provide proactive care beyond the point of crisis [29]. To support practitioners to deliver this intervention, we emphasise the importance of ensuring there is supervision to enable them to feel confident in working in this way, which is often quite different from the usual role of mental health practitioners in liaison psychiatry teams.

We must acknowledge the 39% of patients who did not take up any of the follow-up sessions on offer in phase 1 and 34% who did not receive any follow-up sessions in phase 2. Anecdotally, we are aware that some practitioners were more flexible in arranging appointments with patients (e.g. offering different options for day, time and mode), which may have helped with engagement. We have made concerted efforts to support patients’ engagement in the sessions, with researchers arranging sessions and sending reminders to patients. Despite this, a substantial number of patients did not attend any sessions. We also attempted to follow up on these patients, including for qualitative interviews, yet it proved difficult to contact them so little is known about why these patients did not attend any sessions. It is possible they may have felt they did not need it, or they may have felt the session would serve as a reminder of their self-harm and ED presentation, which can be a traumatic experience. This is an area for further exploration, to better understand how engagement can be supported.

Findings from phase 1 revealed that aside from recruitment, no other major barriers were encountered with regard to data collection and follow-ups. Importantly, we demonstrated we could feasibly extract primary efficacy outcome data—reattendance to the ED for self-harm—from hospital electronic records. However, given that hospital presentations are considered the ‘tip of the iceberg’ of self-harm episodes [40], we sought to obtain self-report self-harm data monthly via an online survey sent by email. This was to try to obtain a more complete picture of the extent to which patients were self-harming over the follow-up period. However, self-reporting in this way suffered from substantial missing data. Anecdotally, a number of patients reported these emails had gone to their junk emails which may in part explain the missing data. There is a need for improved methods to obtain self-harm data, highlighting the importance of innovation in Ecological Momentary Assessment (EMA) methods such as data collection via text messages and Apps, which may offer better modes of contact than email surveys [41]. A text message survey was developed for the trial with the aim of mitigating some of the issues encountered by collecting data via an online survey sent by email. However, due to the recruitment issues in the internal pilot, we are yet to see whether this improves data collection rates. We acknowledge that self-harm behaviour does not reflect what is important from the patient’s perspective, and therefore, our future trial will incorporate a process evaluation to fully explore the perspectives on their experiences of the intervention and if and how it has led to meaningful change.

In phase 1, completion rates of the research assessments were 87% post-ED (in the days following attendance) and 66% at 6 months. In phase 2, the completion rate for research assessment 1 was 79%. This demonstrates issues with attrition, although this is in line with expected follow-up rates based on other trials in hospital settings [42]. Overall, patients described the research procedures as acceptable. Some changes were made to the measures between phases 1 and 2 to improve the acceptability of the research assessments based on feedback that the measures could be overwhelming. In particular, the C-SSRS was replaced with a briefer measure of suicidality, the BSS, which we anticipate improving the acceptability of the research assessments in the full trial. Patients reported positive experiences of taking part in the study and their interactions with researchers, who were professional and helpful and supported them to answer the questionnaires, particularly the more difficult ones which asked about self-harm and suicide. These are important findings that may help allay fears of conducting sensitive research on the topic of suicide [43].

While some practitioners reported feeling uncomfortable with their clinical work being recorded in the feasibility study, patients did not report concerns about their intervention sessions being recorded, as found in previous research where biopsychosocial assessments in the ED were recorded [9]. This is an important finding, as concerns about the ethics of recording clinical interactions are often raised by ethics committees and clinicians. This study highlights that patients are often happy to be recorded, understanding the value that this can have for improving clinical interactions with mental health practitioners.

Findings show that we could obtain ED reattendance data for self-harm from hospital records without attrition, demonstrating the feasibility of this approach for obtaining primary efficacy outcome data. Over the 6-month follow-up period, reattendance rates were 20% in TAU and 13% in the intervention arm [42].

Findings also showed positive therapeutic alliance scores in both the intervention and TAU arms, and good experiences with general ED staff and liaison psychiatry teams, which appeared to reflect more positive experiences in contrast to the many studies where issues with ED care for mental health have been reported [7, 9, 44].

### Limitations

There were several limitations to this study. Firstly, the primary efficacy outcome was repeat attendance to the hospital for self-harm. This was obtained from the hospital where patients attended, but it is unknown whether patients may have attended other EDs during the follow-up period. We also acknowledge issues that reattendance may not be a meaningful outcome to patients, as reattendance shows help-seeking, which will often reflect patients following advice on their care/safety plan [45]. Further research is needed to identify outcome measures that reflect meaningful change for patients who have previously presented to the ED with self-harm. Process evaluations embedded within trials are one important way of capturing the helpful and hindering factors of such interventions and will be incorporated into our future RCT [46].

While qualitative findings indicate the acceptability of the research procedures, we acknowledge these are based on participants who were available and willing to be interviewed and cannot be assumed to be representative of those who were unable or unwilling to be interviewed.

Owing to the time constraints of consenting patients before their biopsychosocial assessment in the ED, we were unable to collect baseline data for the sample. The feasibility study was mostly conducted when researchers were not on site, due to the pandemic, which meant we were unable to fully test the recruitment methods we intended to use in the internal pilot trial.

The study took place in a very challenging context during the COVID-19 pandemic. Staff workload was high, with high levels of staff sickness, and numerous challenges with staff being redeployed to manage the pandemic. While this made the delivery of the study challenging, the teams were committed to delivering the study reflecting a drive to improve care for this underserved population.

### Next steps for the Assured trial

Our revised trial design moves from randomisation of practitioners to patient randomisation to mitigate some of the challenges in the internal pilot. Eligible patients would be randomised after they are seen by the liaison practitioner for their biopsychosocial assessment, either in the ED or soon after they leave. As such, we would not be reliant on ED and liaison staff to screen/consent patients. This design would enable us to approach all eligible participants presenting to the ED rather than relying on when ED research nurses/researchers and study practitioners are on-site. This is expected to remove the major barriers in recruiting patients, as we will be able to screen all patients presenting to the ED with self-harm and/or suicidal ideation, regardless of who is on-site and whether a study practitioner has conducted their biopsychosocial assessment.

This has implications for the intervention. In its original design, the first intervention components (narrative interview and safety plan) were delivered in the biopsychosocial assessment in the ED. These components will now be delivered in the first follow-up session approximately a week after their ED presentation, with three solution-focussed sessions taking place thereafter. This means that patients in the intervention arm would still receive the intervention, but at over a different timescale. We will remove the 72-h check-in phone call from the intervention, as it will not be feasible to include this within the revised timescale for consenting patients. This modified trial design seeks to account for the realities of the ED context while enabling us to test the intervention in a way that we anticipate being viable to implement into more routine practice if effective. Contamination is not expected to be an issue as patients are not routinely offered follow-up care after seeing liaison psychiatry teams.

There were challenges in getting staff off shift for training and supervision, so ensuring sufficient resources and being able to accommodate the working patterns and rotas (including night shifts) in this future RCT will be essential.

## Conclusion

The feasibility study supported further evaluation of the Assured intervention to reduce reattendance to EDs for self-harm and/or suicidal ideation. However, our internal pilot trial revealed substantial issues with our recruitment procedures. The revised trial design will involve approaching and randomising patients after their biopsychosocial assessment and delivering the intervention soon after someone presents to the ED. The study methods were acceptable to practitioners and patients, the intervention was successfully implemented by practitioners and the majority of patients engaged in it.

## Supplementary Information

Supplementary Material 1.

## Data Availability

Data, intervention materials and the study protocols will be made available upon reasonable request.

## Abbreviations

A&E: Accident and emergency
BSS: Beck Scale for Suicide Ideation
CORE-OM: Clinical Outcomes in Routine Evaluation–Outcome Measure
C-SSRS: Columbia–Suicide Severity Rating Scale
ED: Emergency department
MANSA: Manchester Short Assessment of Quality of Life
RCT: Randomised controlled trial
SIX: Social Outcomes Index
TAU: Treatment as usual
WEMWBS: Warwick-Edinburgh Mental Wellbeing Scales
