# Supplementary material for: Enhanced psychosocial assessment and rapid follow-up care for people presenting to emergency departments with self-harm and/or suicidal ideation: the Assured feasibility study and internal pilot trial
Source: Pilot Feasibility Stud. 2025 Feb 20;11:20. doi: 10.1186/s40814-025-01602-y (PMC11841326; doi:10.1186/s40814-025-01602-y)
Supplement: Supplementary file 1 — Supplementary Material 1. [file 40814_2025_1602_MOESM1_ESM.docx]

**Supplementary Appendix 1. Measures collected at each time point (Phase 1)**

| **Outcome** | **Post-ED** | **Month 1** | **Month 2** | **Month 3** | **Month 4** | **Month 5** | **Month 6** |
| --- | --- | --- | --- | --- | --- | --- | --- |
| Sociodemographics | X |  |  |  |  |  |  |
| Helping Alliance Scale | X |  |  |  |  |  | X |
| Columbia–Suicide Severity Rating Scale (C-SSRS) | X |  |  |  |  |  | X |
| Clinical Outcomes in Routine Evaluation – Outcome Measure (CORE-OM) | X |  |  |  |  |  | X |
| Social Outcomes Index (SIX) | X |  |  |  |  |  | X |
| Manchester Short Assessment of Quality of Life (MANSA) | X |  |  |  |  |  | X |
| Experience in A & E | X |  |  |  |  |  |  |
| Self-reported self-harm (online survey sent by email) |  | X | X | X | X | X | X |
| Client Service Receipt Inventory (CSRI) | X |  |  |  |  |  | X |
| Repeat hospital presentation for self-harm |  |  |  |  |  |  | X |
| Death by suspected suicide |  |  |  |  |  |  | X |

**Supplementary Appendix 2. Measures used in internal pilot trial (Phase 2)**

| **Outcome** | **Post-ED** | **Month 1** | **Month 2** | **Month 3** | **Month 9** | **Month 18** |
| --- | --- | --- | --- | --- | --- | --- |
| Sociodemographics | X |  |  |  |  |  |
| Helping Alliance Scale | X |  |  | X |  |  |
| Beck Scale for Suicide Ideation (BSS) | X |  |  | X | X | X |
| Clinical Outcomes in Routine Evaluation – Outcome Measure (CORE-OM) | X |  |  | X | X | X |
| Social Outcomes Index (SIX) | X |  |  | X | X | X |
| Warwick-Edinburgh Mental Wellbeing Scales (WEMWBS) | X |  |  | X | X | X |
| Experience in A & E | X |  |  |  |  |  |
| Self-reported self-harm (text message survey) |  | X | X | X | X | X |
| Client Service Receipt Inventory (CSRI) | X |  |  | X | X | X |
| Repeat hospital presentation for self-harm |  |  |  |  |  | X |
| Death by suicide |  |  |  |  |  | X |

**Supplementary Appendix 3**

***ASsuRED Case Report Form: Follow up Questionnaire***

Thank you for agreeing to complete this follow-up questionnaire. We are interested to know how you have been in the last month.

*Please note that the information you provide is confidential and will only be used for research purposes.*

Yes No

- 1. **During the past month, have you tried to end your life?**
  2. **During the past month, have you self-harmed?**

***If no to 1.2, end of questionnaire***

***If yes to 1.2, please answer:***

Yes No

- 1. **Did you seek medical help for self-harm or overdose?**
     1. **If yes, please describe where you sought medical help and what treatment you received?**
  2. **How did you self-harm :**

| **Have you self-harmed by:** | **Tick all that apply in the past month** | **Approximately, how many days in the past month have you self-harmed?**  **Please say number of days for each method below.** |
| --- | --- | --- |
| **Restricting consumption of:** |  |  |
| Food  Medication  Other |  |  |
|  |  |  |
|  | (Please state) |  |
| **Ingesting (swallowing):** |  |  |
| Drugs or alcohol  Medications or tablets  Other substances  Other |  |  |
|  |  |  |
|  |  |  |
|  | (Please state) |  |
| **Injuring your skin by:** |  |  |
| Cutting  Scratching/ picking  Burning  Other |  |  |
|  |  |  |
|  |  |  |
|  | (Please state) |  |
| **Injuring your head or body by:** |  |  |
| Banging your head  Hitting/ punching  Other |  |  |
|  |  |  |
|  | (Please state) |  |
| Other: Please state ………………………………………………… |  |  |
| Other: Please state ………………………………………………… |  |  |
| Other: Please state ………………………………………………… |  |  |

- 1. **How do you feel you are coping with/managing your self-harm? Please describe in your own words.**

**Thank you for completing the survey. We value your feedback about how things are going for you.**

**Supplementary Appendix 4**

**Experiences of care in Accident & Emergency (A&E)**

It would be helpful for us to understand more about your experiences in with the general staff you saw in A&E and then the mental health professional you saw in A&E. It will help us know what is done well and what can be improved.

| 1 | 2 | 3 | 4 | 5 | 6 |
| --- | --- | --- | --- | --- | --- |
| Strongly disagree | Disagree | Slightly disagree | Slightly agree | Agree | Strongly agree |

Using the scale above, please select the number that best fits your experience with the ***general staff in A&E who you met before you spoke with the mental health professional.***

| 1. The A&E environment made me feel worse. |  |
| --- | --- |
| 2. My problems were considered to be less important than other people’s physical problems. |  |
| 3. A&E staff explained what was going to happen to me while I was in A&E. |  |
| 4. I was watched too closely by A&E staff. |  |
| 5. A&E staff took my mental distress seriously. |  |
| 6. During any physical contact with me, the A&E staff were kind and caring. |  |
| 7. I felt negatively judged or discriminated against by A&E staff. |  |

Using the scale above, please select the number that best fits your experience with the ***mental health professional in A&E*** ***who spoke to you in detail about your mental health****.*

| 8. The mental health professional listened carefully to what I said. |  |
| --- | --- |
| 9. The room was comfortable and private. |  |
| 10. Our talk was more about ticking boxes than understanding my problems. |  |
| 11. The mental health professional took my distress seriously. |  |
| 12. I found the advice helpful. |  |
| 13. My next steps after leaving A&E were easy to understand. |  |
| 14. I felt negatively judged or discriminated against by the mental health professional. |  |
| 15. The mental health professional cared about me. |  |

Please tick the responses that applies to you:

| 16a. Did you go to A&E with someone like a family member or friend? |  |
| --- | --- |
| No |  |
| Yes |  |
| 16b. If yes, were they included in conversations with the mental health professional **(please tick)**: | |
| Not enough |  |
| About the right amount |  |
| Too much |  |

17. If you would like to say more about your experiences in A&E, please use the space below. We want to hear about any good experiences as well as anything which was unhelpful or upsetting for you.

______________________________________________________________________________________________________________________________________________________________________________________________________________________________________________________________________________________________________________________________________________________________________________________________________

**Supplementary Appendix 5**

**Interview schedule: Practitioner interview**

| - Introduce self - Aim of the interview: - Confidentiality, check participant is still happy to participate in the research - Stress the following points: - We are interested in what is important to you - There are no right or wrong answers, it is your perspective that we are interested in - You are not obliged to answer; if you don't want to talk about a subject, please say so and will move on   Prompts to check participant is happy to explore further sensitive issues: *Is it alright if we talk about that?* |
| --- |

I would like to ask you about your experience of delivering the ASSuRED intervention to people recruited into the study.

**Feasibility of intervention**

1. Could you tell me about how you have found delivering the intervention with people who have presented with self-harm?

- What worked well?
- What didn’t work so well?

*Make sure every component of the intervention is covered:*

- Narrative interview – how did it compare to the usual assessment?
- Safety plan
- 72-hour phone call
- Solution focused follow-ups
- Handover of the safety plan
- Letters

1. Did you include a trusted other in any of the sessions? Explore
2. How did you organise the logistics of the follow ups? (E.g. scheduling, format – intervention interval input from researchers) Challenges / ways to support
3. Were the sessions face-to-face or remote? Explore
4. How did you feel being recorded?

**Delivering intervention within NHS context**

1. How was it different with different patients? And different presenting problems? *E.g. CAMHS/adult/complex cases/people who didn’t engage*
2. How did you find delivering the intervention in the context of the NHS system and other services?

Prompts: Did you have participants who were linked with secondary services? How did that work? What were the challenges? How did you overcome them?

Explore: L*inks with GP’s, communication with other teams e.g. crisis teams/secondary services/voluntary*)

1. Were there challenges in delivering the intervention within the team/organisation?
2. What has supported your delivery of the intervention (personally, within the team, in the organisation)
3. What aspects of the intervention would you change? In what way?

**Training and supervision**

1. How did you find the training? Did you feel ready to deliver the intervention after the training?
2. How did you find the manual?
3. How have you found supervision? Explore: content, frequency, preferences

**Influence on practice**

1. Are there ways in which your practice has changed in working with people presenting with self-harm since completing the training?
2. What would you continue doing in your practice from this new approach?
3. Is there anything else that we haven’t covered that you would like to share about your experience of taking part in the ASsuRED study?

**Interview schedule: Patient interview**

| - Introduce self - Aim of the interview: - Confidentiality, check participant is still happy to participate in the research - Stress the following points: - We are interested in what is important to you - There are no right or wrong answers, it is your perspective that we are interested in. - You are not obliged to answer; if you don't want to talk about a subject, please say so and will move on   General guidance:   - Prompts to check participant is happy to explore further sensitive issues: *Is it alright if we talk about that?* - Mirror wording used by the person - Useful prompts:   • Can you give me an example of…  • Could you describe X further?  • What was the most helpful thing about X?  • What was it that gave you that feeling? |
| --- |

I would like to ask you about your visit to the ED on (date), how the meeting with the mental health practitioner went and about any contacts you have had with the ED team since.

**ED ASSESSMENT**

- Could you tell me about your meeting with the mental health practitioner in the A&E on (date)?
- What worked well for you? What didn’t work so well for you? *(Ask for specific examples)*

***Narrative interview***

- Did you feel you were able to speak openly about what happened to bring you to A&E?

***Question design/communication***

- How relevant were the questions that the practitioner asked you?
- When [practitioner] was asking questions, was there anything in particular you noticed about the way he asked you questions? The way he/she phrased things?

***Safety plan***

- Did you talk about a safety plan? If so, can you tell me about it?
- Were there parts of the safety plan that were helpful?
- Were there parts of the safety plan that were unhelpful?
- Have you used the safety plan since leaving the ED? How?
- Have you received a safety plan from the ED before? If so, how does it compare?

***Evaluating ED assessment***

- What was the most important outcome of the meeting for you?
- Was there anything they didn’t suggest that may have been helpful for you?
- If relevant: How did your experience with this practitioner compare with previous experiences in the ED?

**FOLLOW-UP CONTACTS**

- Have you had any contact or follow-up meetings with the same practitioner you met in the ED?

***IF NO:***

- Explore – what happened? Anything that could have made it possible for them to attend?

***IF YES:***

- Can you tell me about those meetings/calls?
- Did you discuss your best hopes? How did you feel about what was discussed?
- What was helpful/not helpful?
- Did you review the safety plan?
- Did you discuss resources outside of the NHS?
- What did you think about the timing of the follow up sessions? Were they too soon? Too late?
- Did you miss any meetings? Could you tell me about that?
- How did you feel about the follow-up meetings coming to an end?
- Did you discuss your care after the end of the follow-up meetings? *Explore handover of care*.

What was the most important outcome of the follow up sessions for you?

***Delivery of sessions***

- How did your sessions take place (f2f, phone, video)? Would you have preferred a different approach?

**RELATIONSHIP**

- What was your impression of the practitioner in your first meeting?
- How do you feel about your relationship with the practitioner?

How did you feel about meeting with the same practitioner that you met in the ED?

**INVOLVEMENT OF OTHERS**

- Was a trusted other involved in any meetings with you?

***IF YES:***

- Who? How were they involved? How did you feel having them involved?

***IF NO:***

- Would you find it helpful to have someone involved in the meeting with you? How?

**LETTERS**

- Did you receive any letters from the practitioner? If yes: can you tell me what you thought about them?

**CRISES/ SUPPORT SINCE LEAVING ED**

- Have you experienced any periods of distress/crisis since participating in the study? How have you managed them?
- Have you been back to the ED? *If so, explore.*
- Have you seen a GP or other mental health practitioner about self-harm? What happened?

**EXPERIENCE OF TAKING PART IN RESEARCH**

- How did you find taking part in the research? Explore completing measures, sessions being recorded, receiving postcard
  - What do you think of the questions we ask you during the research assessment/ our conversation about your mental health.
  - Do you think the questions capture changes in your mental health?
  - What do you think has changed in your life as a result of taking part in the study?

**Supplementary Appendix 6**

***Fidelity rating scale – ED sessions***

|  | **Done** | **To some extent** | **Not done** |
| --- | --- | --- | --- |
| **ED session** |  |  |  |
| ***Narrative interview (rate items 1-6 based on first 10-15 mins only)*** |  |  |  |
| 1. Used narrative interview opening |  |  |  |
| 1. Used narrative interview techniques to encourage the person to tell their story |  |  |  |
| 1. Shuts the person’s story down |  |  |  |
| 1. Validates the persons distress |  |  |  |
| 1. Start talking about solutions to problems |  |  |  |
| 1. Questions or dispute the person’s story |  |  |  |
| ***Understanding*** |  |  |  |
| 1. Normalises the person’s experience |  |  |  |
| ***Safety plan*** |  |  |  |
| 1. Introduced the safety plan and its purpose |  |  |  |
| 1. Asked about warning signs |  |  |  |
| 1. Asked about distractions |  |  |  |
| 1. Asked about changing environment |  |  |  |
| 1. Asked about people they trust |  |  |  |
| 1. Asked about professionals |  |  |  |
| 1. Identifies someone to share the safety plan with |  |  |  |

***Fidelity rating scale – Follow up sessions***

|  | **Done** | **To some extent** | **Not done** |
| --- | --- | --- | --- |
| 1. Encourages exploration of the persons ‘best hopes’ |  |  |  |
| 1. Encourages exploration what’s already working or exploration of change |  |  |  |
| 1. Explores how they will notice future/further signs of progress |  |  |  |
| 1. Reviews safety plan |  |  |  |
| 1. Explores resources that may be helpful for the person |  |  |  |

**Supplementary Appendix 7**

***ASsuRED electronic-Case Report Form (e-CRF) – Text Messages***

**Opening Message:**

**“Dear Participant, thank you for taking part in the ASSURED study and agreeing to complete this follow-up questionnaire. We are interested to know how you have been in the last month. Please note that the information you provide is confidential and will only be used for research purposes. To STOP receiving text messages for the follow-up questionnaires, reply STOP.”**

**Question 1:**

1. **Have you self -harmed in the past month.**

**If patient response is:**

**Unclear**

**No**

**Yes**

**Then text response is Question 2 / or:**

**I'm sorry, I can't understand your response. Please give your answer as either 'Yes' or 'No'.**

**Thank you!**

**2) How many times? (Please give a number)**

**If patient response is:**

**Not a numerical response**

**A numerical response**

**Then text response is:**

**I'm sorry, I can't understand your response. Please give your answer as a numerical digit (e.g. '1', '2')**

**Thank you!**
